# Structural, magnetic and electrical properties of a new double-perovskite LaNaMnMoO_6_ material

**DOI:** 10.1098/rsos.170920

**Published:** 2017-11-08

**Authors:** Sameh Megdiche Borchani, Wissem Cheikh-Rouhou Koubaa, Makrem Megdiche

**Affiliations:** 1 Institut Supérieur d'Informatique et de Multimédia de Sfax, Pôle technologique de Sfax, BP 242, Sakiet Ezzit, 3021 Sfax, Tunisia; 2 Laboratoire de Caractérisation Spectroscopique et Optique des Matériaux, Sfax University, BP 1171, 3000 Sfax, Tunisia; 3 Laboratoire de Physique des Matériaux, Faculté des Sciences de Sfax, Sfax University, BP 1171, 3000 Sfax, Tunisia; 4 Centre de Recherche en Informatique Multimédia et Traitement Numérique des Données, Technopôle de Sfax, BP 275, Sakiet Ezzit, 3021 Sfax, Tunisia

**Keywords:** double-perovskite, critical exponents, magnetocaloric effect, magnetoresistance, percolation model

## Abstract

Structural, magnetic, magnetocaloric, electrical and magnetoresistance properties of an LaNaMnMoO_6_ powder sample have been investigated by X-ray diffraction (XRD), magnetic and electrical measurements. Our sample has been synthesized using the ceramic method. Rietveld refinements of the XRD patterns show that our sample is single phase and it crystallizes in the orthorhombic structure with Pnma space group. Magnetization versus temperature in a magnetic applied field of 0.05 T shows that our sample exhibits a paramagnetic–ferromagnetic transition with decreasing temperature. The Curie temperature *T*_C_ is found to be 320 K. Arrott plots show that all our double-perovskite oxides exhibit a second-order magnetic phase transition. From the measured magnetization data of an LaNaMnMoO_6_ sample as a function of the magnetic applied field, the associated magnetic entropy change |−ΔSM| and the relative cooling power (RCP) have been determined. In the vicinity of *T*_C_, |−ΔSM| reached, in a magnetic applied field of 8 T, a maximum value of ∼4 J kg^−1^ K^−1^. Our sample undergoes a large magnetocaloric effect at near-room temperature. Resistivity measurements reveal the presence of an insulating-metal transition at Tρ = 180 K. A magnetoresistance of 30% has been observed at room temperature for 6 T, significantly larger than that reported for the A_2_FeMoO_6_ (A = Sr, Ba) double-perovskite system.

## 1. Introduction

Perovskite and double-perovskites oxides with general formula ABO_3_ and A_2_B′B′′O_6_ respectively (A is an alkaline-earth or rare-earth metal ion; B and (B′/B′′) are transition metals) are of significant interest because of the diverse properties exhibited by them. Their properties include colossal magnetoresistance [1–7], large magnetocaloric effects (MCEs) [8–12], multiferroicity [13], magnetodielectric behaviour [14,15] and large magneto-optic responses [16]. In double perovskites, rock-salt ordering of B′ and B′′ ions can be achieved for large size and valence difference between B′ and B′′ cations [17].

Recently, Kobayashi *et al*. [18] reported large, low-field, tunnelling-type magnetoresistance even at room temperature in ordered double perovskites A_2_B′B′′O_6_. The most ordered double perovskite studied to date is Sr_2_FeMoO_6_ [19–23]. It is known that FeO_6_ and MoO_6_ octahedra are alternately ordered in a rock-salt lattice and the angle of the Fe–O–Mo chain is nearly 180°. This oxide is a half metal and shows a ferrimagnetic ordering behaviour at low temperature with a high ordering temperature *T*_C_* *∼ 420 K, in which spins at the Fe^3+^(3*d*^5^) and Mo^5+^(4*d*^1^) ions are aligned in the opposite direction. However, this homologous manganese double-perovskite Sr_2_MnMoO_6_ with Mn^2+^(3*d*^5^) and Mo^6+^(4*d*^0^) ions has been known to be either a paramagnetic (PM) or antiferromagnetic insulator [24]. Hence, the Fe- and Mn-compounds show opposite magnetic behaviours in Sr_2_(Fe/Mn)MoO_6_. The same result has been observed recently in an NaLaB′B′′O_6_ (B′ = Mn, Fe, B′′ = Nb, Ta) double-perovskite system [25]. The magnetic interactions inside Mn-compounds are ferromagnetic (FM) in nature, but antiferromagnetic for Fe-compounds. The magnetism of A_2_B′B′′O_6_ (A = Ca, Sr; B′ = Mn, Fe; B′′ = Ta, Nb) would be a good reference to those NaLaB′B′′O_6_ compounds, and indeed, in other words, the short-range order of Na and La in A site has no/slight effect on the magnetism, in comparison to the perovskites with Ca and Sr in the A site.

The availability of four distinct cation sites in A′A′′B′B′′O_6_ opens the door to the design of magnetic materials with novel topologies. Furthermore, compounds with this ordered double-perovskite-type show the potential for multiferroic behaviour. However, few of the compounds reported in the literature have not been thoroughly examined, and it was partially the purpose of this work to find a novel compound with useful properties.

In the present work, we have prepared, to our knowledge for the first time, and characterized a new member of the Mn-based materials A′A′′MnMoO_6_ family, the LaNaMnMoO_6_ double-perovskite oxide. Then we report a giant magnetoresistance and moderate MCE over a broad temperature range.

## 2. Experimental

Powder samples of LaNaMnMoO_6_ were prepared using the standard ceramic processing technique by mixing La_2_O_3_, Na_2_CO_3_, MnO_2_ and MoO_3_ up to 99.9% purity in the desired proportion according to the following reaction:

$$0.5\text{L}{\text{a}}_{2}{\text{O}}_{3}+0.5\text{N}{\text{a}}_{2}\text{C}{\text{O}}_{3}+\text{Mn}{\text{O}}_{2}+\text{Mo}{\text{O}}_{3}\to \text{LaNaMnMo}{\text{O}}_{6}+\delta \text{C}{\text{O}}_{2}.$$

The starting materials were intimately mixed in an agate mortar and then heated in air at about 950°C for 72 h with intermediate regrinding. A systematic annealing at high temperature is necessary to ensure a complete reaction. In fact, the powder sample is pressed into pellets (of about 1 mm thickness) and sintered at 1100°C in air for 48 h with intermediate regrinding and repelling. Finally, these pellets were rapidly quenched at room temperature in air.

Phase purity, homogeneity and cell dimensions were determined by powder X-ray diffraction (XRD; X-ray powder SIEMENS diffractometer) at room temperature using Fe radiation. The structure refinement was carried out using the Rietveld technique [26]. The energy-dispersive X-ray analysis was performed using a scanning electron microscope (SEM) (Philips FEI QUANTA 200). DC magnetization of the sample was recorded in the Faraday balance in the temperature range 50–350 K. Magnetization at various fields was measured using a vibrating sample magnetometer in fields up to 8 T. Resistivity measurements as a function of the temperature and the applied magnetic field were carried out on dense ceramic pellets by the standard four-probe technique.

## 3. Results and discussion

### 3.1. Crystal structure

**Figure 1.**
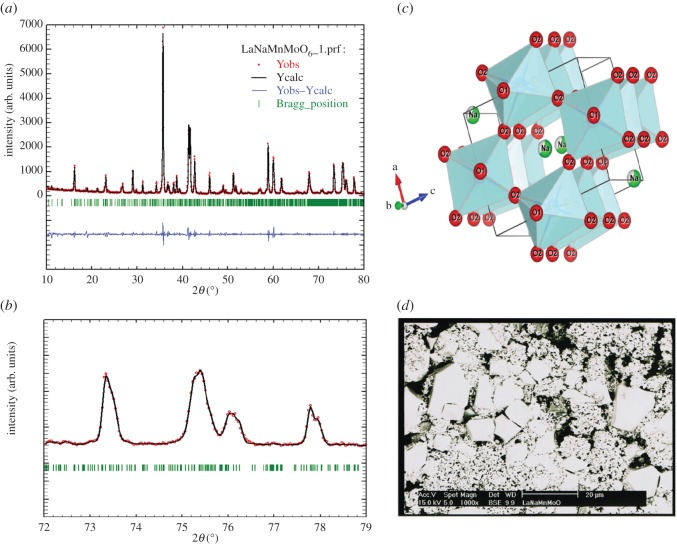
Typical powder X-ray diffraction (XRD) profile (*a*), the fit between the observed and the calculated profiles (*b*), the schematic view of the orthorhombic crystal structure (*c*), and the SEM images (*d*) of the LaNaMnMoO_6_ sample. Dots and solid line represent the observed and calculated profiles, respectively. The difference plot is drawn below the profile, and vertical bars represent the allowed reflections.

Powder XRD patterns indicate that our synthesized LaNaMnMoO_6_ sample is single phase. No impurity has been detected. Structure refinements using the Rietveld method have been achieved with the Fullprof program [27]. Figure 1*a* shows the XRD patterns (measured, calculated and Bragg reflection positions) at room temperature for LaNaMnMoO_6_. A good fit between the observed and the calculated profiles was obtained (as indicated in figure 1*b*). All the peaks are indexed in the orthorhombic system with Pnma space group. The structural parameters obtained from the Rietveld refinement of the XRD pattern at room temperature and the selected bond distances with angles of the LaNaMnMoO_6_ sample are listed in tables 1 and 2, respectively. The cell lattice sets are ∼√2*a*p × 2*a*p × √2*a*p, where *ap* is the unit cell parameter of the parent perovskite aristotype. The same symmetry and the same order of magnitude for the volume of the elementary cell were obtained for its homologous oxides LaNaB′B′′O_6_ (B′ = Mn, Fe, B′′ = Nb, Ta) by Dachraoui *et al.* [25]. Moreover, our samples present a ratio c/a < √2 (table 1) characteristic of a cooperative Jahn–Teller deformation. The strong orthorhombic distortion of the LaNaMnMoO_6_ structure is because of the cooperative coupling of the MnO_6_ and MoO_6_ Jahn–Teller distorted octahedral B sites.

**Table 1.** Structural parameters obtained from the Rietveld refinement of the XRD pattern at room temperature and atomic position of the LaNaMnMoO_6_ with Pnma space group.

atom	Wyckoff	*x*	*y*	*z*	Occ	Ueq
Na	4c	0.0135	0.25	−0.0041	0.73	0.15
La	4c	0.0135	0.25	−0.0041	0.27	0.15
Mn	4b	0.00	0.00	0.5	0.26	0.0061
Mo	4b	0.00	0.00	0.5	0.72	0.061
O1	4c	0.4937	0.25	0.0697	1	0.0043
O2	8d	0.2710	0.4710	0.7257	1	0.009
a (Å), 4.5420; b (Å), 8.6958; c (Å), 6.2654; volume (Å^3^), 247.4602; *χ*^2^, 2.020; R_p_, 1.607%; R_wp_, 1.689%

**Table 2.** Selected bond distances (Å) and angles (°) in LaNaMnMoO_6_.

NaLaMnMoO_6_	
Na/La-O (Å)	−O1/2.44660
	−O1/2.27352
	−O2/2.98787*2
	−O2/2.63919*2
	−O2/2.80315*2
〈Na/La-O〉 (Å)	2.6975
Mn/Mo-O (Å)	−O1/1.56077*2
	−O2/2.32082*2
	−O2/2.10585*2
〈Mn/Mo-O〉 (Å)	1.9958
Mn/Mo-O1-Mn/Mo (°)	134.4794
Mn/Mo-O2-Mn/Mo (°)	169.2965

Figure 1*c* shows the orthorhombic structure of LaNaMnMoO_6_ projected along the (101) direction, where the (Mn/Mo)O_6_ octahedra are apparent. A typical feature of the crystal structure of these double-perovskite oxides is the presence of a superlattice owing to the ordered arrangement of the cations in the oxygen octahedral nodes (B sites). The superlattice formation owing to displacement of the anions from their ideal sites may be also considered as another cause.

The SEM image of the LaNaMnMoO_6_ ceramic is presented in figure 1*d*. A larger grain (≈3.5 µm) with well-defined boundaries, which coexist with smaller ones, was noted for the LaNaMnMoO_6_ ceramic. The average crystallite size can be evaluated from the width of diffraction peaks using Scherrer formula [28]:

3.1 $$C_{\mathrm{XRD}}=\frac{K\lambda }{\beta \cos\theta },$$

where *K* is the grain shape factor, *λ* is the X-ray wave length, and *θ* and *β* are the Bragg angle and the width at half maximum of the XRD peak, respectively. The C_XRD_ of LaNaMnMoO_6_ powder is found to be 41.57 nm. Obviously, the grain sizes observed by SEM are several times larger than those calculated by XRD, which indicates that each grain observed by SEM is composed of several crystallites.

The ideal structure of the double perovskites is based on the adapted tolerance factor *t* of the single perovskite [29]. In general, for double perovskites A_2_B′B′′O_6_, the tolerance factor can be written as [30] follows:

3.2 $$t=\frac{\langle r_{A}\rangle +r_{O}}{\sqrt{2}(\langle r_{B}\rangle +r_{O})},$$

where *r*_A_, *r*_B_ and *r*_O_ are the average ionic radii of the A site, B site and oxygen, respectively. The closer to *t* = 1, the more the structure corresponds to ideal cubic. Therefore, except in rare cases, one can consider the following rule for the double-perovskite family: for 1.05 > *t* > 1.00, a cubic structure is adopted within the space group; for 1.00 > *t* > 0.97, the most likely structure corresponds to the I 4/m tetragonal space group and if *t* < 0.97, the compound becomes either monoclinic (*P*21/*n*) or orthorhombic [31]. Table 3 shows the evolution of the tolerance factor of our sample LaNaMnMoO_6_ and of its homologous double-perovskite oxide LaNaB′B′′O_6_/A_2_MnMoO_6_ with different symmetry. These values are in good agreement with those mentioned below. Our crystallographic data were used to also calculate this tolerance factor for LaNaMnMoO_6_ such as

3.3 $$t=\frac{\left\langle A-O\right\rangle }{\surd 2\langle B-O\rangle },$$

where A–O and B–O are the bond length (Na/La)–O and (Mn/Mo)–O, respectively.

**Table 3.** Tolerance factor with crystal symmetry of our powder LaNaMnMoO_6_ and of their homologous samples LaNaB′B′′O_6_/A_2_MnMoO_6_.

A′A′′B′B′′O_6_/A_2_B′B′′O_6_	symmetry	tolerance factor *t*	reference
Ba_2_MnMoO_6_	Fm-3 m	1.009	[32]
LaNaMgTeO_6_	P4/mmm	0.971	[33]
LaNaMnMoO_6_	Pnma	0.969	this work
LaKMnMoO6	P222	0.964	[34]
Sr_2_MnMoO_6_	P21/n	0.952	[32]
NaLaMgWO_6_	C2/m	0.94	[33]
NaLaZr_2_O_6_	Pnma	0.93	[33]
LaNaMnWO_6_	P21/m	0.926	[35]

This factor is 0.956 which is close to the Goldschmidt one (equation (3.2)).

### 3.2. Magnetic properties

**Figure 2.**
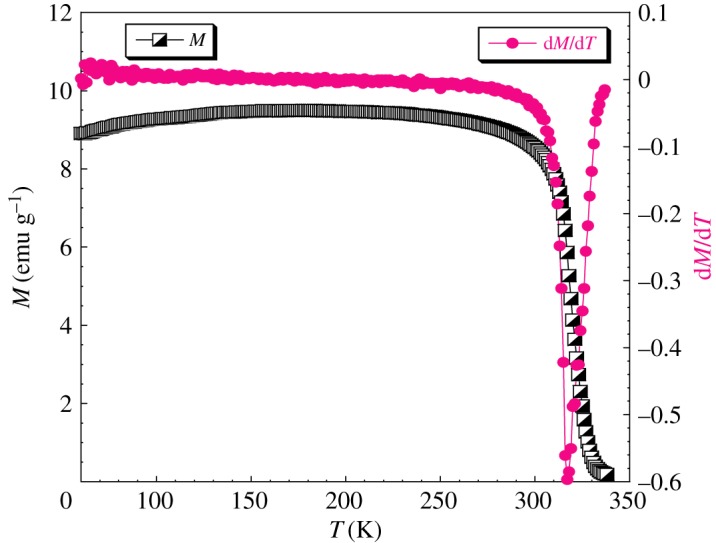
Magnetization and the d*M*/d*T* (T) curve of LaNaMnMoO_6_ as a function of temperature at *H* = 500Oe.

A previous study shows that LaKMnMoO_6_ compound exhibits an FM behaviour at low temperature. The Curie temperature *T*_C_ is 180 K [34]. Magnetization versus temperature for the LaNaMnMoO_6_ sample are plotted in figure 2. Our synthesized sample LaNaMnMoO_6_ exhibits a PM to FM transition at *T*_C_ = 320 K with decreasing temperature. *T*_C_ has been determined from the peak position of the d*M*/d*T* curve, as shown in figure 2. Changing potassium content to sodium does not destroy the FM behaviour observed in LaKMnMoO_6_ compound at low temperature; however, it induces an increase in the Curie temperature *T*_C_ from 180 K for LaKMnMoO_6_ to 320 K for LaNaMnMoO_6_. This result can be explained by the decrease of the average ionic radius $\left\langle r_{A}\right\rangle$ of the A cation site of A′A′′MnMoO_6_ double-perovskite samples and/or the distortion of the octahedral MnO_6_ and MoO_6_ as observed in the Pr_0.7_Ba_0.3−x_MnO_3_ perovskite sample [36]. The FM behaviour increases with decreasing the average ionic radius $\left\langle r_{A}\right\rangle$ from 1.500 Å with *T*_C_ = 180 K for the LaKMnMoO_6_ double-perovskite sample to 1.375 Å with *T*_C_ = 320 K for LaNaMnMoO_6_.

Our oxide is FM at low temperature. Such FM behaviour has not been observed in this homologous Sr_2_MnMoO_6_ sample [24].

FM–PM transition of LaNaMnMoO_6_ was modelled using a phenomenological model given by Hamad [37]. The dependence of magnetization on the variation of temperature is written by

3.4 $$M=\frac{\left(M_{i}-M_{f}\right)}{2}\tanh(A^{*}T_{C}-T))+B^{*}T+C,$$

— *M*_i_/*M*_f_ is an initial/final value of magnetization at an FM–PM transition;
— *B* is the magnetization sensitivity d*M*/d*t* at FM state before transition;
— *A* = 2*(*B* − *S*_C_)/(*M*_i_ − *M*_f_);
— *S*_C_ is the magnetization sensitivity d*M*/d*T* at Curie temperature *T*_C_; and
— *C* = (*M*_i_ − *M*_f_)/2 − B*T_C_.

**Figure 3.**
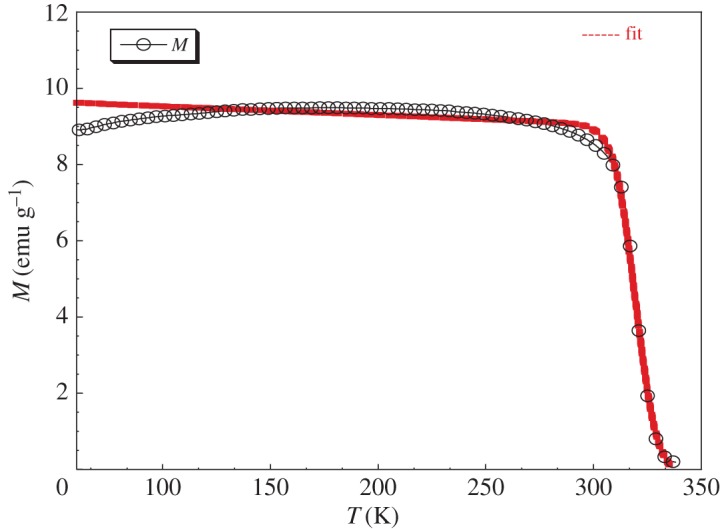
Magnetization versus temperature for the LaNaMnMoO_6_ sample at 50 mT magnetic field. The solid line represents modelled result and the symbol represents experimental data.

Fitting the *M*(T) measurements of our powder LaNaMnMoO_6_ by the expression (3.4) based on this phenomenological model shows a good concordance between theoretical and experimental study (figure 3). Model parameters for the LaNaMnMoO_6_ sample under magnetic field 0.05 T are listed in table 4.

**Table 4.** Model parameters for the LaNaMnMoO_6_ sample in 500 Oe applied magnetic field of *M*(T) curve.

*μ*_0_*H*(T)	(*M_i_* − *M_f_*)/2 (emu g^−1^)	*T*_C_ (K)	*S*_C_ (emu g^−1^ K^−1^)	*B* (emu g^−1^ K^−1^)
0.050	4.5980	319.54	−0.6	−0.00231

**Figure 4.**
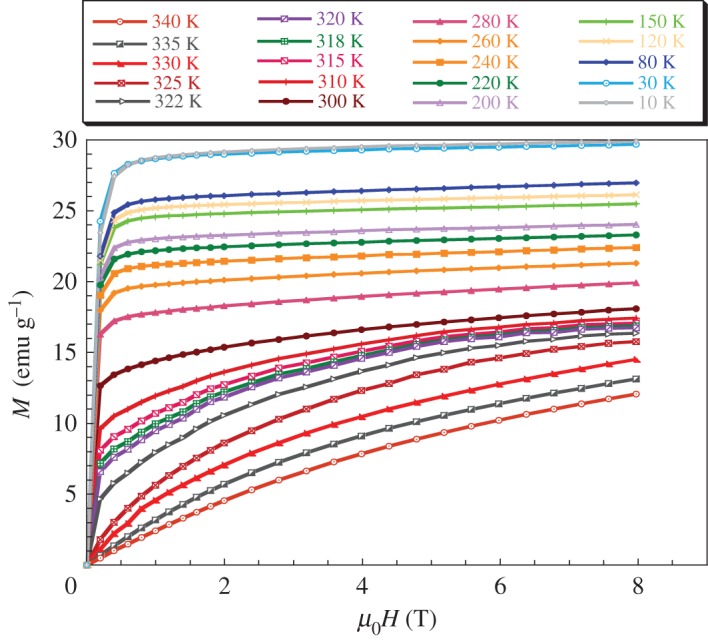
Magnetization *M* versus magnetic applied field *µ*_0_*H* up to 8 T at several temperatures for LaNaMnMoO_6_ powder.

In order to confirm the FM behaviour at low temperatures of our sample, we performed magnetization versus magnetic applied field up to 8 T at several temperatures. We plot in figure 4 the *M* (*μ*_0_*H*) curves for LaNaMnMoO_6_ compound. The magnetization rises sharply for low magnetic applied field and then saturates for *µ*_0_*H* higher than 1 T. The saturation magnetization at 10 K and 5 T, deduced from the *M* (*μ*_0_*H*) curve, is 2.1 µ_B_/mole. The LaNaMnMoO_6_ compound reaches approximately 50% of the theoretical saturation of Mn3+ (4 µB for *S* = 2).

**Figure 5.**
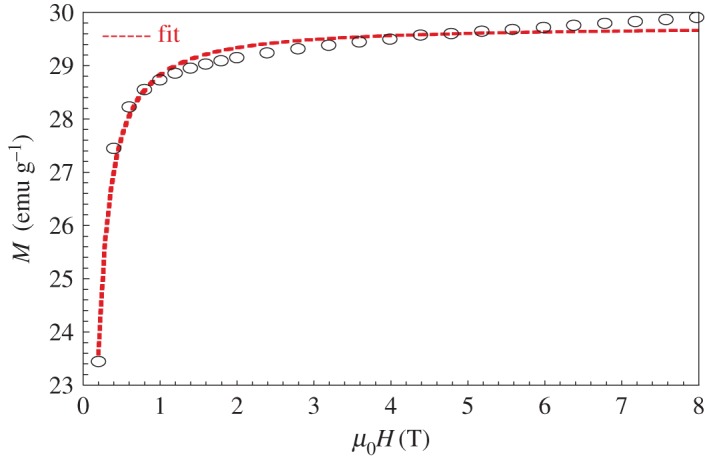
The simulated curve *M* (*μ*_0_*H*) at 10 K for the LaNaMnMoO_6_ sample.

The *M* (*μ*_0_*H*) curves can be simulated by the law-approach to saturation (figure 5) in the term [38]:

3.5 $$M={M_{S}}^{*}\left(1-\frac{a}{{\left(\mu_{0}H\right)}^{n}}\right),$$

where 0 ≤ *n* ≤ 1, *M*_s_ is the saturation of magnetization, (*a*/(*μ*_0_*H*)*^n^*) indicates the deviation of magnetization from saturation and the factor *n* changes with respect to the origin of deviation. The *a*-factor correlates with the FM correlation length. The values of *a*, *n* and *M*_s_ are given in table 5. The large *n* factor and the small *a* factor for the compound should be associated with the long-range spin order of magnetic moment.

**Table 5.** Simulated parameters of *M*(*μ*_0_*H*) at 10 K for the LaNaMnMoO_6_ sample.

LaNaMnMoO_6_	
$M_{s}^{\mathrm{fitt}}\;({\mathrm{emu}\;g}^{-1})$	29.743(0)
a	0.031(0)
n	1.176(7)

**Figure 6.**
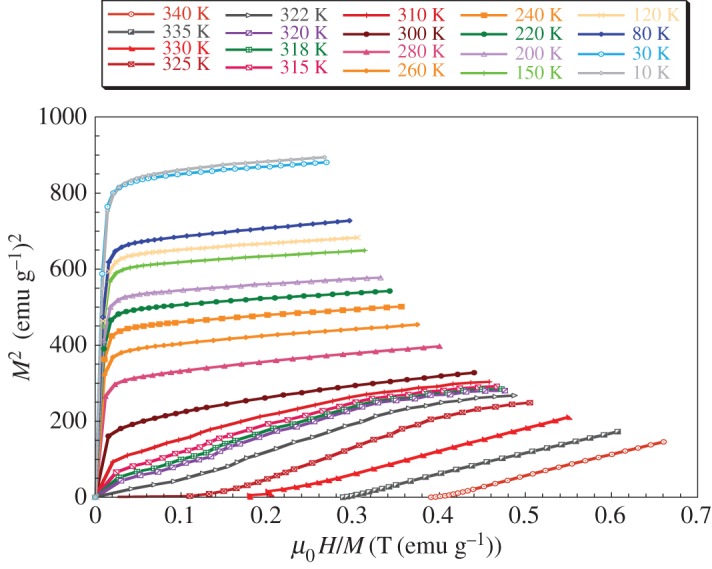
Arrott curves (*M*^2^ versus *µ*_0_*H*/*M* isotherms) for LaNaMnMoO_6_.

We report in figure 6, the Arrott curves *M*^2^ versus *μ*_0_*H*/*M* for the LaNaMnMoO_6_ sample. All the *M*^2^ versus *μ*_0_*H*/*M* curves clearly show a positive slope for the complete temperature range, which means that a second-order FM to PM phase transition occurs (according to the criterion proposed by Banerjee [39]). The *T*_C_ values deduced from the Arrott curves are very close to those obtained from the *M*(T) curve (*T*_C_ = 320 K).

**Figure 7.**
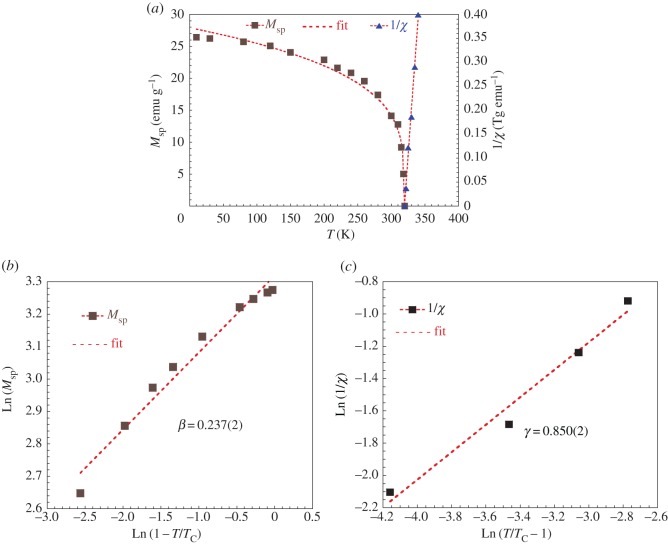
(*a*) Spontaneous magnetization and inverse of susceptibility versus T for LaNaMnMoO_6_ compound with simulation (red straight line). (*b*) The Ln–Ln plot used to determine the critical exponent *β*. (*c*) The Ln–Ln plot used to determine the critical exponent *ϒ*.

Figure 7*a* shows the temperature dependence of the spontaneous magnetization and the inverse of the magnetic susceptibility evolution versus temperature for LaNaMnMoO_6_ compound.

We have simulated our experimental measurements of spontaneous magnetization and the inverse of susceptibility versus temperature by theoretical expression based on Bloch's Law and Curie Weiss Law, respectively. We have obtained a best fit between experimental and theoretical study. Parameters fit are listed in table 6.

**Table 6.** Parameters fit of spontaneous magnetization and the inverse of susceptibility versus temperature.

Bloch's Law: *M*_sp _= *M*_0_ (1 − (*T*/*T*_C_))^*β*			Curie Weiss Law: 1/*χ* =−(*T*_C_/*C*)+(1/*C*)*T*	
*M*_0_ (emu g^−1^)	*T*_C_ (K)	*β*	−(*T*_C_/*C*) (T g emu^−1^)	1/*C* (T g emu^−1^ K^−1^)
27.769(1)	319.986(0)	0.243(6)	6.176(1)	0.019(3)

The effective magnetic moment ($\mu_{\mathrm{eff}}^{\exp}$) can be estimated using simple Curie Weiss formula:

3.6 $$\chi =\frac{C}{T-T_{C}},$$

where $C={\left(\mu_{\mathrm{eff}}^{\exp}\right)}^{2}\text{/}8$ [40].

From the determined (*C*) parameter, we have deduced the experimental effective moment: $\mu_{\mathrm{eff}}^{\exp}=5.547\;{\text{μ}}_{B}$. Compared to the theoretical value calculated considering all the magnetic species inside the structure, both the Mn^3+^ (3d^4^) and Mo^5+^ (4d^1^) ions contribute to the PM behaviour and the effective magnetic moment of the compounds, ($\mu_{\mathrm{eff}}^{\mathrm{th}}$) is given by the following equation: $\mu_{\mathrm{eff}}^{\mathrm{th}}=\sqrt{\mu_{\mathrm{eff}}^{2}(\text{M}{\text{n}}^{3+})+\mu_{\mathrm{eff}}^{2}(\text{M}{\text{o}}^{5+})}=5.196\;{\text{μ}}_{B}$. The observed discrepancy between both values indicates that in the PM state, the spins do not exist as individuals; they are rather assembling in small groups revealing the presence of the FM correlations in the PM phase.

**Figure 8.**
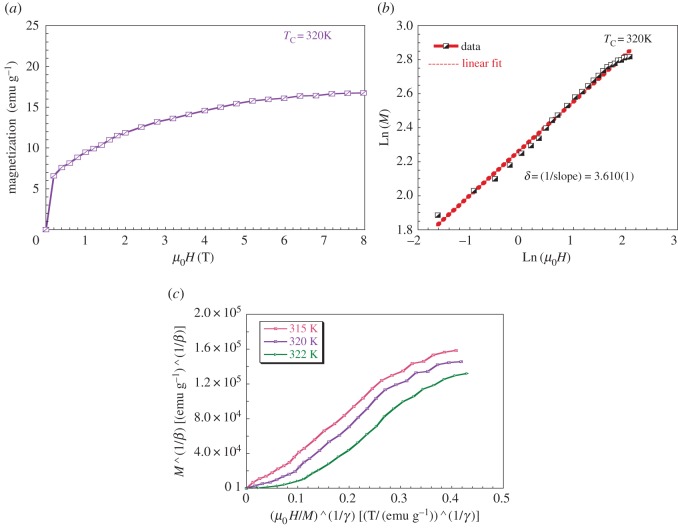
(*a*) *M* versus *µ*_0_*H* isotherm measured at *T*_C_ = 320 K. (*b*) The Ln–Ln plot for the critical exponent *δ* calculation for LaNaMnMoO_6_ compound. (*c*) The modified Arrott plot for LaNaMnMoO_6_ compound.

The calculated value of the critical exponent *β* is obtained from fitting Ln(*M*_sp_) versus Ln((*T*_C_ − *T*)/*T*_C_) plot to a be straight line (figure 7*b*). Similarly, Ln (1/*χ*) versus Ln ((*T* − *T*_C_)/*T*_C_) plot allows the determination of *ϒ* (figure 7*c*). The *β* and *ϒ* values are 0.237(2) and 0.850(2), respectively. Using these critical exponents, we have represented the modified Arrott plots for LaNaMnMoO_6_ compound, as shown in figure 8*a*. These values are close to those expected for the tricritical mean-field theory model (*β* = 0.25, *ϒ* = 1) [41]. Moreover, *δ* exponent can be determined by fitting the Ln–Ln plot of *M* (*μ*_0_*H*) curve at *T*_C_ (figure 8*b*,*c*). The determined value of *δ* = 3.61 for the LaNaMnMoO_6_ sample is smaller than is calculated from the Widom relation [38]: *δ* = 1 + (*ϒ*/*β*) = 4.586; this difference is related to the existence of the magnetic inhomogeneity (Griffiths Clusters) in the vicinity of the transition temperature.

### 3.3. Magnetocaloric characterizations

A giant field-induced entropy change is one of the important criteria for magnetocaloric materials. In fact, the isothermal measurements of magnetization allow us to determine the magnetic entropy change of the sample under an applied magnetic field, according to the classical thermodynamic theory based on Maxwell's relations using the following equation:

3.7 $$\Delta S_{M}(T,H)=\sum_{i}\frac{M_{i+1}(T_{i+1},H)-M_{i}(T_{i},H)}{T_{i+1}-T_{i}}.$$

**Figure 9.**
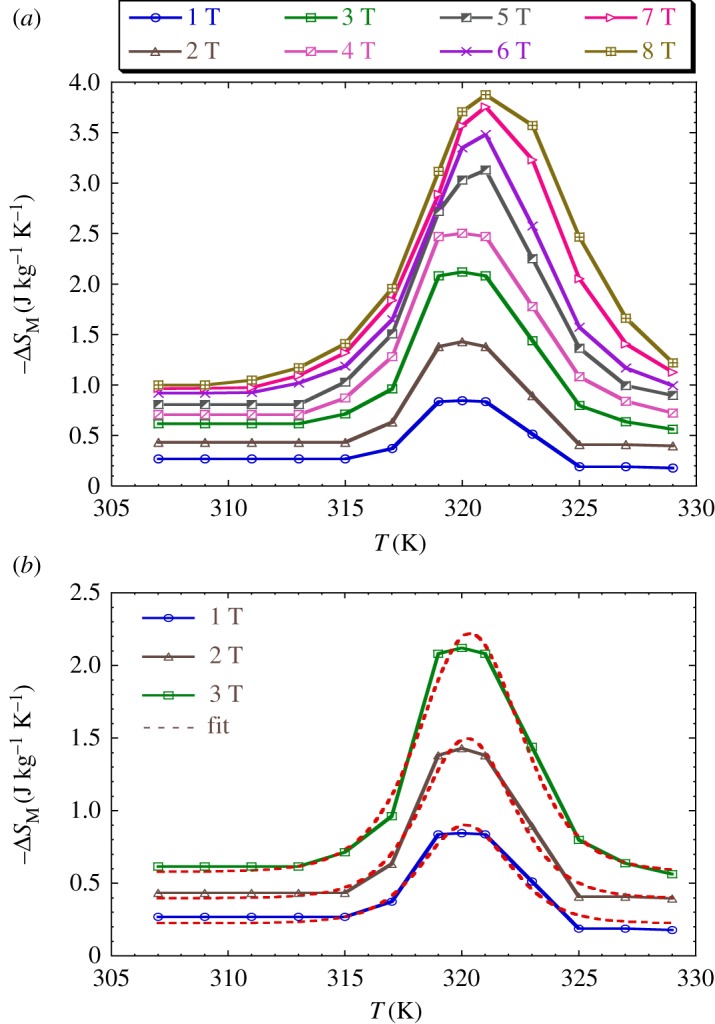
(*a*) Magnetic entropy change versus temperature under various magnetic applied field changes. (*b*) The plot fit (red line) of magnetic entropy change versus temperature for 1 T, 2 T and 3 T according to the percolation model (via equation (3.8)).

*M_i_* and *M_i_*_+1_ are the experimental values of the magnetization measured at temperatures *T_i_* and *T_i_*_+1_, respectively, under an applied magnetic field *H*. The magnetic entropy change can be measured through either the adiabatic change of temperature by the application of a magnetic field, or through the measurements of classical *M*(*H*) isotherms at different temperatures [42]. In our work, we have used the second method based on magnetization measurements versus magnetic field. Figure 9*a* shows the behaviour of the magnetic entropy change as a function of temperature under several values of external magnetic field for our double-perovskite sample.

According to the phenomenological model [43], the magnetic entropy change of a magnetic system under adiabatic magnetic field variation from 0 to final value *H*_max_ is available by

3.8 $$\Delta S_{M}=\left[-A\left(\frac{M_{i}-M_{f}}{2}\right)\text{sec}{\text{h}}^{2}(A(T_{C}-T))+B\right]H_{\max}.$$

Using the above equation, we numerically calculated the variation of the magnetic entropy (Δ*S*_M_) depending on the temperature of our sample (figure 9*b*). The theoretical and experimental results are illustrated in table 7 under a magnetic field of 1 T, 2 T and 3 T. A good concordance is observed.

**Table 7.** Parameters fit for the LaNaMnMoO_6_ sample in 1 T, 2 T and 3 T applied magnetic field of (−Δ*S_M_* (T)) curves.

*μ*_0_*H*(T)	*A* (emu g^−1^ K^−1^)	(*M*_i_ − *M*_f_)/2 (emu g^−1^)	*T*_C_ (K)	*B* (emu g^−1^ K^−1^)
1	0.427(1)	0.200(3)	320.270(0)	−0.028(1)
2	0.420(9)	0.331(9)	320.340(0)	−0.049(5)
3	0.393(6)	0.520(8)	320.510(0)	−0.076(8)

Under an applied magnetic field of 2 T, the absolute value of Δ*S* of the LaNaMnMoO_6_ sample is 1.5 J kg^−1^ K^−1^ around *T*_C_, and it reaches 3.99 J kg^−1^ K^−1^ under a magnetic field change of 8 T. Although the Δ*S* values in our compound are smaller than that observed in Gd (Δ*S* = 4.2 J kg^−1^ K^−1^) for Δ*H* = 2 T [41] considered as the best magnetic refrigerant, the LaNaMnMoO_6_ sample can be considered as a potential candidate for magnetic refrigeration.

In order to confirm the important MCE of our specimens, it is interesting to consider the relative cooling power (RCP) which can be determined from the following relation:

3.9 $$\text{RCP}=-\Delta S{\left(T,H\right)}^{*}\delta_{\mathrm{FWHM}}.$$

*δ*_FWHM_ is full width at half maximum of Δ*S*(T) curve [45]. The RCP value is 41.99 J kg^-1^ under an applied magnetic field of 8 T. These results are interesting, compared with those of materials considered as good for applications in magnetic refrigerators. Our sample undergoes a large MCE above room temperature.

**Figure 10.**
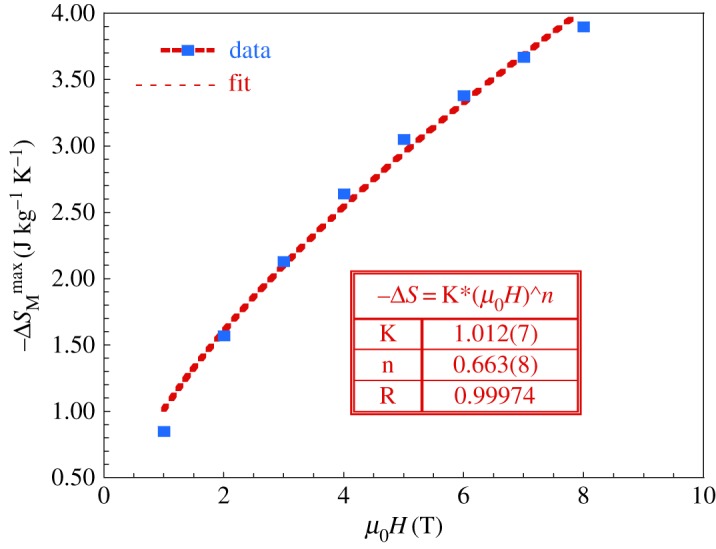
Field dependence of the magnetic entropy change around the transition temperature for LaNaMnMoO_6_. The inset shows the fit parameters of equation (3.10).

The magnetic field dependence of the magnetic entropy change of materials with a second-order phase transition can be expressed as [46]:

3.10 $$\Delta S_{M}^{\mathrm{Max}}\approx {\left(\mu_{0}H\right)}^{n},$$

where *n* depends on the magnetic state of the sample and it is obtained from the fit plot of $\Delta S_{M}^{\mathrm{Max}}$ versus *µ*_0_*H* via equation (3.8) (figure 10). The value of *n* deduced from the fitting is equal to 0.66(5) (inset of figure 10). This value is different from the calculated using the relation;

3.11 $$n=1+\frac{\beta -1}{\beta +\gamma },$$

[47] (*n* = 0.29(8)). This difference shows the signature of magnetic inhomogeneities in our sample.

**Figure 11.**
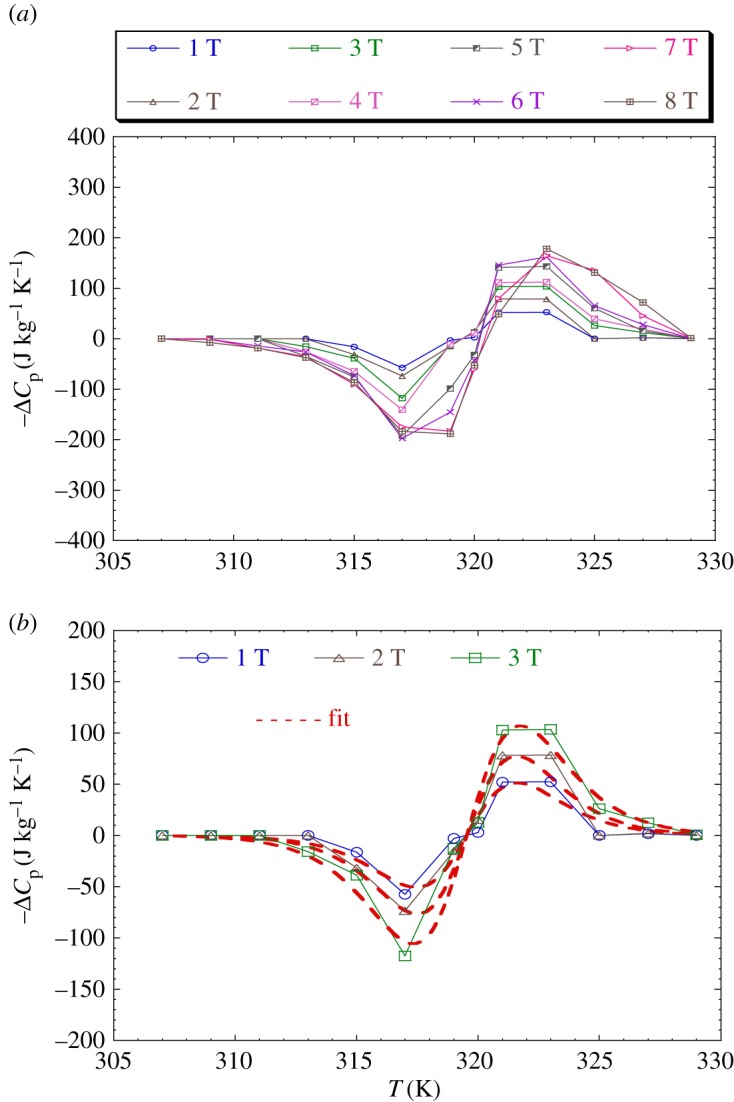
(*a*) Temperature dependence of Δ*C*_p_ under different field variations for the LaNaMnMoO_6_ sample. (*b*) Temperature dependence of calculated Δ*C*_p_ for 1, 2 and 3 T applied magnetic fields via the percolation model (equation (3.11)).

Figure 11*a* shows the temperature dependence of heat capacity Δ*C*_p_ under different field variations in our sample calculated from the Δ*S*_M_ data using the following relation:

3.12 $$\Delta C_{p}=T\frac{\partial \Delta S_{M}}{\partial T}.$$

Δ*C*_p_ presents positive values above *T*_C_ and negative ones below *T*_C_. The maximum/minimum values of Δ*C*_p_, observed at 323/317 K are 52.261/−68.839 J kg^−1^ K^−1^ and 78.630/−150.178 J kg^−1^ K^−1^ under 1 T and 2 T, respectively.

Figure 11*b* shows the calculated Δ*C*_p_ as a function of temperature at 1 T and 2 T, using equation (3.11) below:

3.13 $$\Delta C_{p}=[-TA^{2}(M_{i}-M_{f})\text{sec}{\text{h}}^{2}(A(T_{C}-T))\text{tanh}(A(T_{C}-T))]H_{\max},$$

together with the corresponding experimental data for comparison. The predicted values of temperature dependence of ΔCp under 1, 2 and 3 T magnetic field variations in the LaNaMnMoO_6_ sample are shown in table 8.

**Table 8.** Predicted values of temperature dependence of ΔCp under 1, 2 and 3 T magnetic field variations for the LaNaMnMoO_6_ sample.

*μ*_0_*H*(T)	*A* (emu g^−1^ K^−1^)	(*M*_i_ − *M*_f_) (emu g^−1^)	*T*_C_ (K)
1	0.316(9)	0.492(6)	319.530(0)
2	0.316(1)	0.755(9)	319.95(0)
3	0.304(3)	1.212(4)	320.14(0)

Based on figure 11*b*, a good agreement is observed between experiment and calculation, showing the ability of the phenomenological model in the ordered double-perovskite single-crystal LaNaMnMoO_6_, particularly for low magnetic fields.

### 3.4. Electrical properties

**Figure 12.**
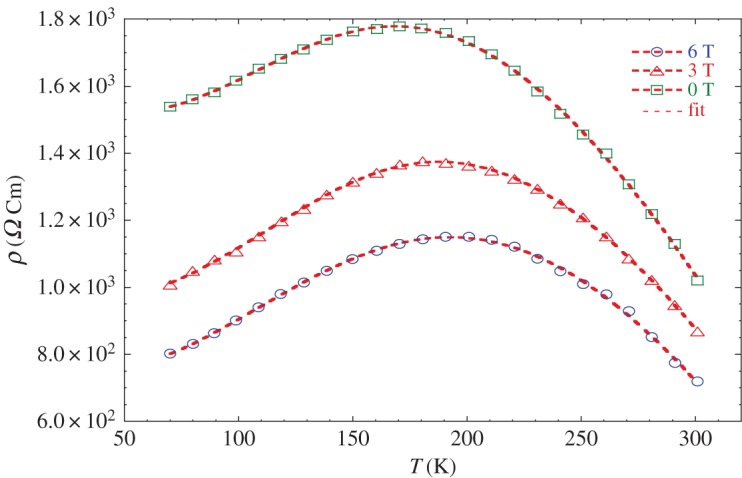
The temperature dependence of resistivity for LaNaMnMoO_6_ under various magnetic fields 0, 3 and 6 T. Symbols are the experimental data and red solid lines are the resistivity calculated using equation (3.14) corresponding to the parameters indicated in table 9.

The temperature dependence of the resistivity *ρ*(T) without and for an applied magnetic field of 3 T and 6 T are plotted in figure 12. The electrical resistivity of LaNaMnMoO_6_ powder attains 1533 Ω cm at 70 K. Then it passes a maximum at *T_ρ_* = 180 K and drops down to 1018.93 Ω cm at room temperature. The magnitude of resistivity is markedly higher than found for Ba_2_YIrO_6_ single crystal (*ρ* (300 K) = 40 mΩ cm) [48].

For comparison, our compound shows that it is metallic at low temperatures (*T* < *T_ρ_*), whereas his homologous Sr_2_MnMoO_6_ sample is an insulator [24].

It should be noted here that there is a large difference between the electric (*T_ρ_* = 180 K) and magnetic (*T*_C_ = 320 K) transition temperature values. The significant difference between *T_ρ_* and *T*_C_ values may be owing to several factors: (i) smaller crystallite size of sample than that measured using XRD, (ii) influence of extrinsic contributions such as a large number of grain boundaries, and (iii) spin-polarized tunnelling between FM grains through an insulating grain boundary layer, and so on. The discrepancy of the grain size may be that the size measured using SEM is for grains consisting of more than one crystallite [49].

To understand the transport mechanism in the whole temperature range, we used the phenomenological percolation model [50,51], which is based on the phase segregation of FM and PM semiconductor regions.

Following this model, we carried out a quantitative analysis of the resistivity temperature dependence data for our sample. According to Li [51], the resistivity for the entire temperature range may then be expressed as follows:

3.14 $$\begin{array}{rl} \rho (T) & =(\rho_{0}+\rho_{2}T^{2}+\rho_{4.5}T^{4.5})\left(\frac{1}{\left(1+\exp(-U_{0}(1-T/T_{C}^{\mathrm{mod}})/k_{B}T)\right)}\right)\\ & \;+\rho_{a}T\exp\left(\frac{E_{a}}{k_{B}T}\right)\left(1-\left(\frac{1}{1+\exp(-U_{0}(1-T/T_{C}^{\mathrm{mod}})/k_{B}T)}\right)\right). \end{array}$$

Based on the phase segregation mechanism (percolation model), the total resistance of the system could be visualized as the sum of the resistivity of the phase separated FM–metallic and PM-insulator: *ρ* = *ρ*_FM*_f + *ρ*_PM*_(1 − *f*); $f=(1/(1+\exp(-U_{0}(1-T\text{/}T_{C}^{\mathrm{mod}})/k_{B}T)))$ is the volume concentration of the FM phase, and (1 − *f*) is the volume concentration of the PM phase.

**Figure 13.**
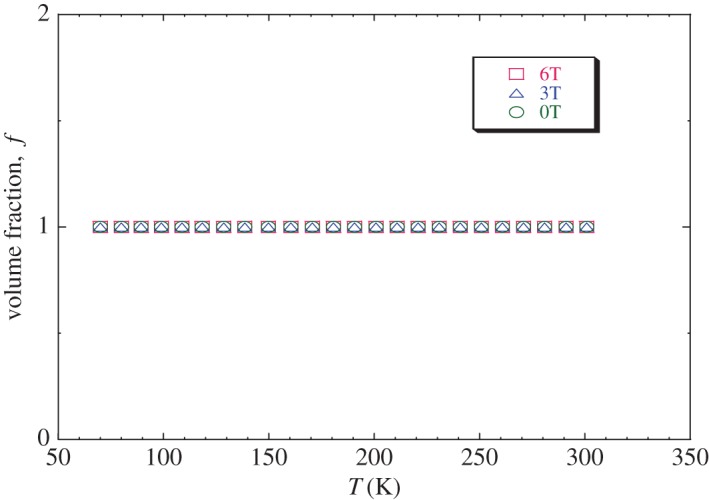
The temperature dependence of ferromagnetic (FM) phase volume fraction *f* (T) for LaNaMnMoO_6_ under applied magnetic fields 0, 3 and 6 T.

In order to see the correlation between the magnetic and electrical properties in our sample LaNaMnMoO_6_, we have fitted the experimental resistivity data (figure 12) using the above equation. It can be seen that the results calculated from equation (3.14) agree with the experimental data. Then, we found that the percolation model describes well enough the resistivity behaviour in a wide temperature range including the region of phase transition, whatever the external magnetic field. The best-fit parameters are given in table 9. The temperature dependence on the volume concentration of the FM phase *f* is shown in figure 13. It is clear that *f* (T) remains equal to 1 below the metal–semiconductor transition temperature, which confirms the strong dominance of the FM fraction in this range.

**Table 9.** Obtained parameters corresponding to the best fit to the equation (3.12) of the experimental data of LaNaMnMoO_6_ at 0, 3 and 6 T.

*μ*_0_*H*(T)	0	3	6
*ρ*_0_ (Ω cm)	1120.7	677.48(3)	543.36(6)
*ρ*_2_ (Ω cm K^2^)	0.017(5)	0.020(9)	0.018(2)
*ρ*_4.5_ (10^−8^ Ω cm^4.5^)	1.052(6)	1.009(6)	0.942(5)
*U*_0_/*K*_B_ (K)	869.97	873.46	863.52
*T*_C_ (K)	321.04	320.08	323.19
*ρ*_a_ (10^−5^ Ω cm)	0.1066(7)	0.077(6)	0.079(7)
*E*_a_/*K*_B_ (K)	955.23	955.34	934.33

**Figure 14.**
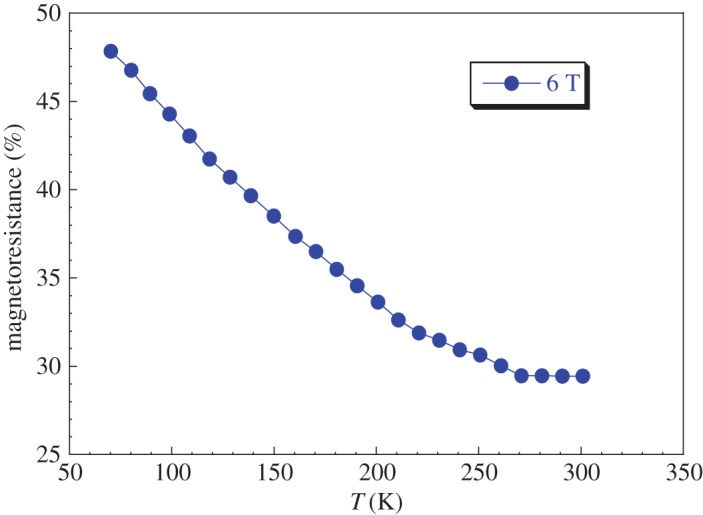
The temperature dependence of magnetoresistance for LaNaMnMoO_6_ under applied magnetic field of 6 T.

The coexistence of ferromagnetism and metallic conductivity at low temperatures provides evidence of the existence of magnetoresistance MR. Defining the MR at a given temperature as MR = Δ*ρ*/*ρ* = ((*ρ*(0) − *ρ*(H))/*ρ*(0))*100; where *ρ*(H) and *ρ*(0) are the resistivity in a magnetic applied field H and in a zero field, respectively. The magnetoresistance evolution versus temperature at the applied magnetic field (6 T) is illustrated in figure 14. The MR increases with decreasing temperature for the LaNaMnMoO_6_ double-perovskite sample. It is found to be approximately 30% at room temperature and approximately 50% at 70 K at 6 T for our synthesized sample. We thus obtain a large magnetoresistance in the LaNaMnMoO_6_ sample as that observed in the Sr_2_FeMoO_6_ compound by Yuan *et al.* [19]. This result (large MR at room temperature and at low magnetic field) is explained by the effect of grain boundaries. This phenomenon was observed in the Sr_2_FeMoO_6_ compound by Kobayashi *et al.* [18].

## 4. Conclusion

We have investigated structural, magnetic, magnetocaloric, electrical and magnetoresistance properties of an LaNaMnMoO_6_ double-perovskite sample. Structure analysis reveals that our sample crystallizes according to the orthorhombic structure with Pnma space group. Magnetic measurements show a PM–FM transition with decreasing temperature. This new double perovskite exhibits an MCE and a large magnetoresistance near room temperature. A combination of both MCE and large MR in LaNaMnMoO_6_ material makes the appropriate substance for magnetic refrigeration applications at room temperature.

## Data accessibility

This article has no supporting data.
